# Effects of Electroencephalographic Biofeedback Therapy on Depression Level, Sleep Quality and Cognitive Function in Patients With Non-Demented Vascular Cognitive Impairment

**DOI:** 10.62641/aep.v53i5.2010

**Published:** 2025-10-05

**Authors:** Li Wu, Li Zhang, Kezheng Du, Xin He, Xuan Wu, Weiwei He, Yi Wu

**Affiliations:** ^1^Department of Neurology, Affiliated Hospital of North Sichuan Medical College, 637000 Nanchong, Sichuan, China; ^2^Department of Rehabilitation, Affiliated Hospital of North Sichuan Medical College, 637000 Nanchong, Sichuan, China; ^3^Department of Ophthalmologic, Affiliated Hospital of North Sichuan Medical College, 637000 Nanchong, Sichuan, China; ^4^Department of Neurosurgery, Affiliated Hospital of North Sichuan Medical College, 637000 Nanchong, Sichuan, China; ^5^Department of Gastrointestinal Surgery II, Affiliated Hospital of North Sichuan Medical College, 637000 Nanchong, Sichuan, China; ^6^Department of General Surgery Il, Guang’an District People’s Hospital of Guang’an City, 638000 Guang’an, Sichuan, China; ^7^Institute of Hepatobiliary, Pancreatic, and Intestinal Diseases, North Sichuan Medical College, 637000 Nanchong, Sichuan, China

**Keywords:** neurofeedback, cognitive dysfunction, depression, sleep quality

## Abstract

**Objective:**

This study aimed to investigate the effects of electroencephalographic biofeedback (EEG-BF) treatment on cognitive function, sleep quality, anxiety and depression levels and quality of life in patients with vascular cognitive impairment-no dementia (VCI-ND).

**Methods:**

This study was a retrospective study that included a total of 128 patients diagnosed with VCI-ND at the Affiliated Hospital of North Sichuan Medical College from July 2022 to July 2024. The patients were divided into an EEG-BF group and a control group in accordance with whether they received EEG-BF treatment or not. Both groups received standard vascular risk factor management. The EEG-BF group separately received EEG-BF intervention two times a week for 12 weeks. Propensity score matching (PSM) was used to perform 1:1 nearest-neighbour matching between the two groups with respect to baseline characteristics. The matching variables included age; education; place of residence; family income; type of health insurance; number of underlying diseases; and pre-intervention scores on the Montreal Cognitive Assessment (MoCA), Pittsburgh Sleep Quality Index (PSQI), Self-rating Anxiety Scale (SAS), and Self-rating Depression Scale (SDS). The main outcome measures were the PSQI, MoCA, SAS, 36-item Short-Form Questionnaire (SF-36) and SDS before and after treatment.

**Results:**

After PSM, the baseline covariates between the two groups were well balanced, with no significant differences. The Love plot showed a significant decrease in standardised differences in covariates after matching. After 12 weeks of intervention, the EEG-BF group was significantly better than the control group in terms of MoCA scores (*p* = 0.013), SAS scores (*p* = 0.002), SDS scores (*p* = 0.004) and some of the SF-36 dimensions, and the within-group before and after comparisons was statistically different (*p* < 0.05). The sleep quality of the EEG-BF group improved after treatment, whereas that of the control group exhibited no notable variation before and after the intervention (*p* > 0.05).

**Conclusion:**

EEG-BF may help improve cognitive function, sleep quality, emotional state and life quality in patients with VCI-ND, offering a promising individualised non-pharmacological intervention for this population. Future multicentre, prospective studies are needed to further validate its prolonged therapeutic effect and neuromodulatory mechanisms.

## Introduction

The burden of cognitive impairment due to cerebrovascular disease is increasing as the global population ages [1, 2]. Vascular cognitive impairment (VCI) encompasses different stages ranging from mild cognitive impairment (MCI) to vascular dementia (VaD) [2]. VaD-no dementia (VCI-ND) represents an early stage of the VCI spectrum that has not yet met the diagnostic criteria for dementia and is usually characterised by a mild decline in attention, verbal fluency, information processing speed or executive function. Epidemiological studies have shown that the prevalence of VCI-ND is significant and particularly high in older adults after haemorrhagic and ischemic stroke [1, 3]. The reported incidence of MCI in the Chinese population aged 65 years and older is 20.8%, with cerebrovascular disease and vascular risk factor-associated MCI accounting for 42% of all MCIs [4]. Although it does not have a serious effect on activities of daily living, available evidence suggests that VCI-ND is an important precursor state for patients to progress to VaD [2]. However, a notable detail that attention to VCI-ND in current clinical practice is still insufficient, interventions are limited and clinical management needs to be optimised.

Patients with VCI are often associated with considerable emotional and sleep problems [5]. Earlier research has demonstrated that the prevalence of depression and anxiety symptoms in this population is much higher than in the general elderly population, with many patients presenting with persistent depressed mood, irritability, anxiety and tension to the point of reaching subclinical or clinical diagnostic criteria [6]. In addition, sleep disturbances are a common comorbidity in patients with VCI-ND, mainly manifested by difficulty falling asleep, sleep maintenance problems and subjective decline in sleep quality, which all show an interactive relationship with cognitive decline [3, 7, 8]. These non-cognitive symptoms not only exacerbate the overall functional burden of patients with VCI-ND but also accelerate cognitive decline through inflammation, cortical excitability alteration and other mechanisms. However, no standardised treatment protocols exist for VCI-ND comorbid with dysphoria and sleep disturbances. Therefore, exploring non-pharmacological therapies may be of some clinical relevance. Previous studies have shown that non-pharmacological therapies, including acupuncture, electro-acupuncture and computerised cognitive rehabilitation, have the potential to improve VCI-ND [9].

Electroencephalographic biofeedback (EEG-BF), also known as neurofeedback, is an intervention technique that is based on real-time EEG signals; it aims to train patients to autonomously regulate specific brainwave activities through operant conditioning mechanisms and thus improve neurological functioning [10]. Recent studies have found that EEG-BF shows potential value in alcohol use disorders, attention-deficit hyperactivity disorder (ADHD), and substance use disorder [10, 11, 12]. The intervention effect of this therapy in psychosomatic disorders, such as anxiety, depression and insomnia, has a positive significance [12, 13, 14, 15]. To date, few investigations have addressed relevant studies on the use of EEG-BF in patients with VCI-ND, especially the limited evidence on the improvement of patients’ cognitive function, emotional state and sleep quality, which urgently warrants further investigation.

This study aimed to systematically investigate the efficacy of EEG-BF in improving cognitive function, depression, anxiety and sleep quality in patients with VCI-ND. The therapeutic potential of EEG-BF in patients with VCI-ND was clarified by retrospectively assessing the changes in key functional indicators before and after treatment, with the aim of providing new and translationally meaningful non-pharmacological treatment strategies for this population and laying the foundation for the application and dissemination of neuromodulation techniques in early intervention of geriatric MCI.

## Methods

### Study Design

This study is a retrospective study of patients who presented to the Affiliated Hospital of North Sichuan Medical College and were diagnosed with VCI-ND between July 2022 and July 2024. VCI-ND is diagnosed in accordance with the Chinese guidelines for the diagnosis and treatment of vascular cognitive impairment, which requires the presence of MCI, characterised by impairment in cognitive domains (language, memory, attention, visuospatial structure, executive function, calculation, abstract thinking or orientation) and, at the same time, cranial MRI or CT examination revealing cerebrovascular lesions consistent with cognitive impairment. In addition, the location and severity of the lesion had a reasonable causal relationship with cognitive decline [4]. The inclusion criteria in the present study were as follows: meeting the diagnostic criteria for VCI-ND and age ≥18 years. The exclusion criteria were as follows: illiteracy, cognitive decline resulting in limitation of activities of daily living, previous definite diagnosis of dementia, comorbid severe mental disorders, presence of severe somatic disorder affecting cognitive assessment (e.g., severe aphasia or hearing impairment), comorbid with other severe primary disorders and severe missing follow-up data or incomplete data on key scales. This study was approved by the Medical Ethics Committee of the Affiliated Hospital of North Sichuan Medical College (2024ER199-1), and all data were extracted from the hospital’s electronic medical record system. In accordance with the Declaration of Helsinki, this study ensured the protection of patients’ rights, privacy, and dignity. Being a retrospective study and all data having been anonymised before analysis, the Ethics Committee agreed that informed consent of patients and their guardians could be waived for this study.

### Treatment

Patients were assigned to either the control group or the EEG-BF group on the basis of the treatment that they received. Whether the patients received EEG-BF treatment or not was selected by the patients themselves and their families in accordance with their own wishes. A total of 128 patients were finally included, with 69 in the control group and 59 in the EEG-BF group. The control group received standard vascular risk factor management and healthy lifestyle interventions, including control of blood glucose, lipid levels and blood pressure in accordance with clinical guidelines. Smoking and drinking restrictions, regular exercise, reasonable diet and other health guidance were provided. Cognitive improvement drugs, such as cholinesterase inhibitors and N-methyl-D-aspartate (NMDA) receptor antagonist, were not routinely used unless clinically necessary, and no new antidepressants or anxiolytics were added. In the EEG-BF group, EEG-BF treatment was added on the above basis. The BBB-1A type brain biofeedback therapeutic instrument produced by Guangzhou Runjie Medical Equipment Co. (Guangzhou City, Guangdong Province, China) was used during the therapeutic process. During the treatment, patients were placed in a sitting or semi-lying position in a quiet and comfortable environment. After scalp cleaning was performed, the electrodes for surface EEG recording were arranged following the International 10–20 configuration. The EEG for 3–5 min was preliminarily recorded, and the amplitude changes in θ, α, SMR, low β bands and high β bands were analysed to determine the training target. For patients with cognitive decline, the main training strategy was to suppress θ wave and high β wave abnormal activity and enhance SMR and low β wave synchronisation. For patients with sleep disorders, the main strategy was to appropriately increase α wave activity and lower high-frequency activation state. During the training process, real-time rewards were given by means of screen animation and sound feedback to guide patients to self-regulate the brain wave and strengthen the brain electrical activity of the target frequency band. The target frequency bands included suppression of excessive θ (4–7 Hz) and high β (22–30 Hz) activity, along with enhancement of SMR (12–15 Hz), low β (15–18 Hz), and α (8–12 Hz) synchronisation, depending on the patient’s baseline EEG pattern. Each training lasted about 30 min, each time was divided into several short periods of time. Appropriate rest can be taken in the middle of the training to reduce fatigue. The training was performed two times a week for 12 weeks. During the training period, the quality of EEG signals and the state of patients were monitored by the same trained therapist, and the training parameters were dynamically adjusted if necessary.

### Data Collection

The general demographic baseline data of patients with VCI-ND were collected, including sex, age, per capita household income, educational level, type of residence, type of health insurance and number of underlying diseases. Educational level was categorised into three levels: college and above, junior and senior high school and primary school. Per capita monthly household income was categorised into three levels: <3000 CNY (417.16 USD), 3001–5000 CNY (417.30–695.27 USD) and >5000 CNY (695.27 USD). The number of underlying diseases was counted in accordance with the type of chronic diseases with which the patients were comorbid. Cognitive performance was measured using the Beijing edition of the Chinese version of the Montreal Cognitive Assessment (MoCA, Cronbach’s alpha = 0.88), with different cut-offs for different literacy levels; A score of 19 was used as the cut-off for MCI screening for patients with primary school education, and a score of 24 was used for patients with junior high school education and above [16, 17, 18]. Sleep quality was assessed using the Pittsburgh Sleep Quality Index (PSQI, Cronbach’s alpha = 0.734) for a total score of 21, with higher scores indicating poorer sleep quality [16, 19, 20]. The Self-rating Depression Scale (SDS, Cronbach’s alpha = 0.811) and the Self-rating Anxiety Scale (SAS, Cronbach’s alpha = 0.78) were used to evaluate the levels of depression and anxiety, respectively, with standardised scores ≥50 indicating the presence of depression or anxiety symptoms and higher scores indicating more severe symptoms [21, 22, 23]. The Short Form-36 (SF-36) questionnaire, which covers various of dimensions, including physiological functioning, mental health and social functioning, was used to assess patients’ quality of life, with each dimension scored on a scale of 0–100 [24, 25].

### Statistical Analysis

Data collection and statistical analysis were performed by two independent researchers to exclude potential bias. Data were analysed using SPSS (version 26.0, IBM Corp., Armonk, NY, USA) and R (version 4.4.3, R Foundation for Statistical Computing, Vienna, Austria). Continuous variables were tested for normality. Variables with a normal distribution were expressed as mean ± standard deviation, with between- and within-group comparisons performed using independent sample *t*-test and paired *t*-test, respectively. Those that did not conform to normal distribution were expressed as M (P25, P75), and a nonparametric test was used (Mann–Whitney U test or Wilcoxon signed-rank test). Frequencies and percentages were used to describe categorical variables, which were compared between groups by using chi-square test. Propensity score matching (PSM) was used for 1:1 nearest neighbour matching between the two groups to reduce the effect of confounders. After PSM, a total of 118 (59 in both groups) patients were statistically analysed. The test level was two-tailed, and statistical significance was set at *p* < 0.05. In addition, variables that remained imbalanced after PSM were further included in the multivariate regression models for sensitivity analysis to assess their potential influence on the outcomes. Prior to model fitting, multicollinearity among covariates was assessed using variance inflation factors (VIFs), and no significant collinearity was observed (VIFs <5).

## Results

### Comparison of Baseline Characteristics Between the Two Groups

In total, 128 patients meeting the criteria for VCI-ND were included, including 59 in the EEG-BF group and 69 in the control group. As shown in Table 1, the analysis of baseline demographic and clinical features in the two groups before PSM revealed that except for a statistical difference in per capita household income (*p* = 0.030), the variables age, sex, educational level, place of residence, medical insurance type, number of chronic diseases, baseline MoCA score, baseline PSQI, baseline SAS and baseline SDS were not significantly different (*p* > 0.05).

**Table 1.** **Baseline characteristics before propensity score matching**.

		Control group (n = 69)	EEG-BF group (n = 59)	Statistic	*p* value
Age, mean ± SD		67.94 ± 6.70	69.08 ± 8.26	t = –0.86	0.389
Sex, n (%)				χ^2^ = 0.29	0.589
	Female	26 (37.68)	25 (42.37)		
	Male	43 (62.32)	34 (57.63)		
Education level, n (%)				χ^2^ = 2.17	0.338
	Primary school	27 (39.13)	17 (28.81)		
	Junior/senior high school	35 (50.72)	32 (54.24)		
	College and above	7 (10.14)	10 (16.95)		
Place of residence, n (%)				χ^2^ = 1.15	0.284
	Rural	31 (44.93)	21 (35.59)		
	Urban	38 (55.07)	38 (64.41)		
Per capita household income, n (%)				χ^2^ = 7.04	0.030
	<3000 CNY (417.16 USD)	23 (33.33)	10 (16.95)		
	3000–5000 CNY (417.30–695.27 USD)	36 (52.17)	31 (52.54)		
	>5000 CNY (695.27 USD)	10 (14.49)	18 (30.51)		
Medical insurance type, n (%)				χ^2^ = 2.50	0.114
	Resident medical insurance	49 (71.01)	34 (57.63)		
	Employee medical insurance	20 (28.99)	25 (42.37)		
Number of chronic diseases, n (%)				χ^2^ = 1.41	0.493
	≤1 chronic disease	17 (24.64)	18 (30.51)		
	1–3 chronic diseases	40 (57.97)	28 (47.46)		
	>3 chronic diseases	12 (17.39)	13 (22.03)		
Baseline MoCA, mean ± SD		19.09 ± 3.71	19.98 ± 3.24	t = –1.44	0.151
Baseline SAS, mean ± SD		54.86 ± 5.39	54.24 ± 5.77	t = 0.63	0.533
Baseline SDS, M (P25, P75)		52.00 (49.00, 54.00)	52.00 (51.00, 54.00)	Z = –0.54	0.592
Baseline PSQI, M (P25, P75)		8.00 (7.00, 10.00)	9.00 (8.00, 11.00)	Z = –1.25	0.212

EEG-BF, electroencephalographic biofeedback; SD, standard deviation; MoCA, Montreal Cognitive Assessment; PSQI, Pittsburgh Sleep Quality Index; SAS, Self-rating Anxiety Scale; SDS, Self-rating Depression Scale.

### Comparison of Baseline Characteristics After PSM in the Two Groups

**Fig. 1.**
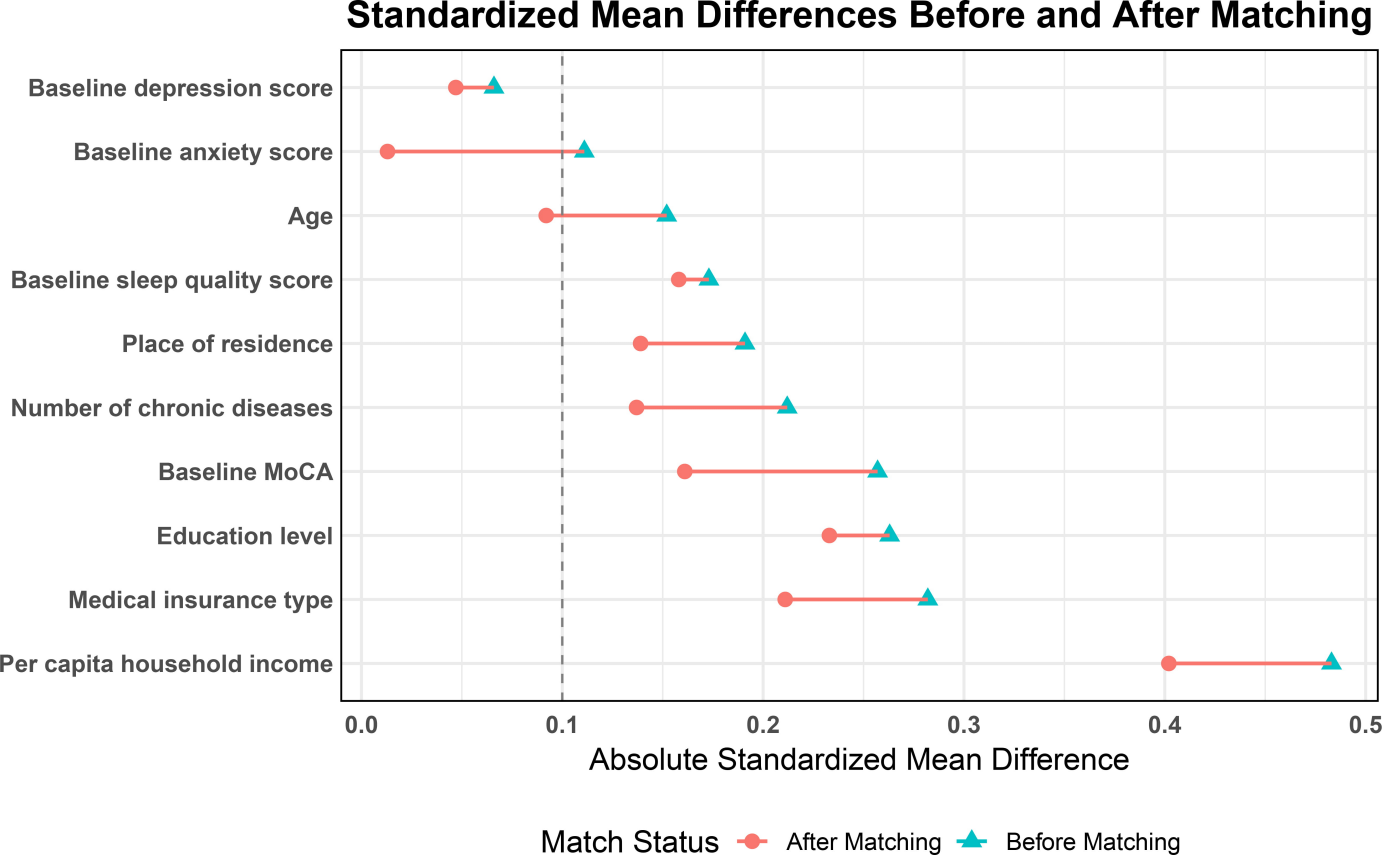
**Standardised mean differences of baseline covariates before and after propensity score matching**. MoCA, Montreal Cognitive Assessment.

PSM was performed on the remaining 10 covariates except sex by using 1:1 nearest neighbour matching to obtain paired samples that were as well balanced as possible on baseline characteristics to control for confounding. As shown in Table 2, after PSM, the EEG-BF and control groups were balanced on baseline characteristics, including per capita household income, which was significantly different before PSM (*p* > 0.05). The quality of PSM matching was further assessed using standardised mean difference (SMD). Table 3 and Fig. 1 demonstrate the change in SMD of each covariate before and after matching. The SMDs of most variables were significantly lower after matching (SMD <0.2). However, a notable detail that the SMDs of per capita household income, medical insurance type and educational level remained above the commonly accepted threshold of 0.2 after matching.

**Table 2.** **Baseline characteristics after propensity score matching**.

Variables		Control group (n = 59)	EEG-BF group (n = 59)	Statistic	*p* value
Age, mean ± SD		68.39 ± 6.86	69.08 ± 8.26	t = –0.50	0.620
Sex, n (%)				χ^2^ = 0.14	0.708
	Female	23 (38.98)	25 (42.37)		
	Male	36 (61.02)	34 (57.63)		
Education level, n (%)				χ^2^ = 1.58	0.455
	Primary school	23 (38.98)	17 (28.81)		
	Junior/senior high school	29 (49.15)	32 (54.24)		
	College and above	7 (11.86)	10 (16.95)		
Place of residence, n (%)				χ^2^ = 0.57	0.450
	Rural	25 (42.37)	21 (35.59)		
	Urban	34 (57.63)	38 (64.41)		
Per capita household income, n (%)				χ^2^ = 4.57	0.102
	<3000 CNY (417.16 USD)	18 (30.51)	10 (16.95)		
	3000–5000 CNY (417.30–695.27 USD)	31 (52.54)	31 (52.54)		
	>5000 CNY (695.27 USD)	10 (16.95)	18 (30.51)		
Medical insurance type, n (%)				χ^2^ = 1.30	0.253
	Resident medical insurance	40 (67.80)	34 (57.63)		
	Employee medical insurance	19 (32.20)	25 (42.37)		
Number of chronic diseases, n (%)				χ^2^ = 0.55	0.759
	≤1 chronic disease	16 (27.12)	18 (30.51)		
	1–3 chronic diseases	32 (54.24)	28 (47.46)		
	>3 chronic diseases	11 (18.64)	13 (22.03)		
Baseline MoCA, Mean ± SD		19.44 ± 3.51	19.98 ± 3.24	t = –0.87	0.384
Baseline SAS, Mean ± SD		54.17 ± 4.94	54.24 ± 5.77	t = –0.07	0.945
Baseline SDS, M (P25, P75)		52.00 (50.00, 54.50)	52.00 (51.00, 54.00)	Z = –0.05	0.963
Baseline PSQI, M (P25, P75)		9.00 (7.00, 10.00)	9.00 (8.00, 11.00)	Z = –1.11	0.269

EEG-BF, electroencephalographic biofeedback; SD, standard deviation; MoCA, Montreal Cognitive Assessment; PSQI, Pittsburgh Sleep Quality Index; SAS, Self-rating Anxiety Scale; SDS, Self-rating Depression Scale.

**Table 3.** **Standardised mean differences of baseline covariates before and after propensity score matching**.

Variable	SMD before PSM	SMD after PSM
Age	0.152	0.092
Education level	0.263	0.233
Place of residence	0.191	0.139
Per capita household income	0.483	0.402
Medical insurance type	0.282	0.211
Number of chronic diseases	0.212	0.137
Baseline MoCA	0.257	0.161
Baseline PSQI	0.173	0.158
Baseline anxiety score	0.111	0.013
Baseline depression score	0.066	0.047

MoCA, Montreal Cognitive Assessment; SMD, standardised mean difference; PSM, propensity score matching; PSQI, Pittsburgh Sleep Quality Index.

### Comparison of MoCA Between the Two Groups

As shown in Table 4, in terms of cognitive function, no significant difference in MoCA scores was observed between the two groups prior to the intervention (*p* > 0.05). After 12 weeks of intervention, the MoCA score of the EEG-BF group was significantly higher than that pre-intervention (*p* = 0.010). The post-intervention score in the intervention group was significantly higher than that in the control group (*p* = 0.013). The control group showed no significant changes in MoCA scores before and after the intervention (*p* > 0.05).

**Table 4.** **Comparison of MoCA scores before and after intervention in two groups**.

		Control group (n = 59)	EEG-BF group (n = 59)	Statistic	*p* value
MoCA, M (P25, P75)					
	Baseline	20.00 (17.00, 22.00)	20.00 (17.00, 23.00)	Z = –1.00	0.318
	Post-treatment	20.00 (17.50, 21.00)	21.00 (18.00, 23.00)	Z = –2.48	0.013
	Statistic	Z = –0.77	Z = –2.58		
	*p* value	0.440	0.010		

MoCA, Montreal Cognitive Assessment; EEG-BF, electroencephalographic biofeedback.

### Comparison of PSQI Between the Two Groups

For the changes in sleep quality, no significant difference was observed in baseline PSQI between the groups (Table 5, *p* > 0.05). The EEG-BF group showed a significant decrease in PSQI after treatment with EEG-BF (*p* < 0.001), whereas the change before and after treatment in the control group was not significant (*p* > 0.05). After the intervention, the EEG-BF group exhibited better sleep scores than the control group, with the difference approaching statistical significance (*p* = 0.076).

**Table 5.** **Comparison of PSQI scores before and after intervention in the two groups**.

		Control group (n = 59)	EEG-BF group (n = 59)	Statistic	*p* value
PSQI, M (P25, P75)					
	Baseline	9.00 (7.00, 10.00)	9.00 (8.00, 11.00)	Z = –1.11	0.269
	Post-treatment	9.00 (8.00, 10.00)	8.00 (7.00, 9.00)	Z = –1.77	0.076
	Statistic	Z = –0.94	Z = –4.06		
	*p* value	0.346	<0.001		

PSQI, Pittsburgh Sleep Quality Index; EEG-BF, electroencephalographic biofeedback.

### Comparison of SAS and SDS Between the Two Groups

Comparisons in terms of emotional state are shown in Table 6. No statistically significant differences can be found in the SAS and SDS scores between the two groups before the intervention (*p* > 0.05). After the intervention, the EEG-BF group showed a significant decrease in SAS (*p* = 0.033) and SDS (*p* = 0.047) scores, and no significant change was observed in the scores of the control group before and after the intervention (*p* > 0.05). The SAS (*p* = 0.002) and SDS (*p* = 0.004) scores were significantly lower in the EEG-BF group than in the control group after the intervention, further demonstrating the potential benefits of EEG-BF in emotion regulation.

**Table 6.** **Comparison of SAS and SDS scores before and after intervention in two groups**.

		Control group (n = 59)	EEG-BF group (n = 59)	Statistic	*p* value
SAS, mean ± SD					
	Baseline	54.17 ± 4.94	54.24 ± 5.77	t = –0.07	0.945
	Post-treatment	55.51 ± 6.50	52.29 ± 4.09	t = 3.22	0.002
	Statistic	t = –1.11	t = 2.18		
	*p* value	0.272	0.033		
SDS, M (P25, P75)					
	Baseline	52.00 (50.00, 54.50)	52.00 (51.00, 54.00)	Z = –0.05	0.963
	Post-treatment	52.00 (50.00, 56.50)	51.00 (47.50, 53.50)	Z = –2.86	0.004
	Statistic	Z = –1.09	Z = –1.98		
	*p* value	0.277	0.047		

SD, standard deviation; SAS, Self-rating Anxiety Scale; SDS, Self-rating Depression Scale; EEG-BF, electroencephalographic biofeedback.

### Comparison of SF-36 Between the Two Groups

In terms of quality of life, as shown in Table 7, no significant difference was observed in the SF-36 scores of the two groups in all dimensions before the intervention (*p* > 0.05). The EEG-BF group, after receiving EEG-BF treatment, scored significantly improved in physical functioning (*p* = 0.022), physical role limitation (*p* = 0.035), vitality (*p* = 0.011), social functioning (*p* = 0.002) and mental health (*p* = 0.003) dimensions compared with the control group.

**Table 7.** **Comparison of SF-36 domain scores before and after intervention in two groups**.

		Control group (n = 59)	EEG-BF group (n = 59)	Statistic	*p* value
Physical functioning, M (P25, P75)					
	Baseline	80.80 (57.25, 88.15)	81.40 (55.35, 93.20)	Z = –0.08	0.933
	Post-treatment	78.20 (61.80, 86.85)	85.00 (66.45, 93.20)	Z = –2.30	0.022
Physical role limitation, M (P25, P75)					
	Baseline	65.70 (53.45, 87.95)	78.80 (51.95, 86.70)	Z = –0.22	0.827
	Post-treatment	78.50 (51.50, 89.00)	82.90 (62.30, 93.35)	Z = –2.11	0.035
Bodily pain, M (P25, P75)					
	Baseline	62.10 (48.55, 89.85)	78.80 (56.20, 90.00)	Z = –0.69	0.487
	Post-treatment	66.90 (57.60, 86.10)	83.00 (56.60, 92.65)	Z = –0.94	0.345
General health, M (P25, P75)					
	Baseline	63.00 (52.75, 77.20)	65.00 (47.95, 82.85)	Z = –0.15	0.882
	Post-treatment	65.40 (57.85, 80.10)	76.20 (51.20, 83.95)	Z = –0.61	0.545
Vitality, M (P25, P75)					
	Baseline	80.80 (55.05, 90.50)	80.20 (57.55, 91.40)	Z = –0.44	0.659
	Post-treatment	66.90 (58.55, 87.55)	90.20 (61.10, 95.80)	Z = –2.54	0.011
Social functioning, M (P25, P75)					
	Baseline	91.60 (81.30, 95.90)	91.40 (87.05, 96.00)	Z = –0.03	0.974
	Post-treatment	89.20 (84.15, 95.20)	94.00 (90.55, 96.65)	Z = –3.03	0.002
Emotional role limitation, M (P25, P75)					
	Baseline	80.90 (70.20, 89.70)	83.60 (68.85, 94.65)	Z = –1.29	0.197
	Post-treatment	82.00 (70.50, 93.25)	88.00 (80.40, 90.00)	Z = –1.33	0.185
Mental health, M (P25, P75)					
	Baseline	76.00 (71.25, 83.20)	77.70 (67.95, 90.50)	Z = –0.22	0.825
	Post-treatment	76.90 (68.80, 86.75)	90.20 (74.50, 93.30)	Z = –2.94	0.003

SF-36, 36-item Short-Form Questionnaire; EEG-BF, electroencephalographic biofeedback.

### Sensitivity Analysis of Post-Intervention Outcomes

Given that the per capita household income, educational level and type of health insurance still differed significantly after PSM (SMD >0.2), sensitivity analyses were performed to the main post-treatment outcome indicators to further control for potential confounders. Multiple linear regression models were used, with each post-intervention score as the dependent variable; the intervention group as the main independent variable, and educational level, per capita household income, and type of health insurance included in the model for covariate adjustment. As shown in Table 8, the MoCA scores in the intervention group tended to increase compared with those in the control group after the intervention (β = 0.77, *p* = 0.072). The PSQI scores decreased significantly (β = –0.83, *p* = 0.030), and a significant decrease was found in the SAS and SDS scores (β = –3.22, *p* = 0.002 and β = –2.49, *p* = 0.002). In the SF-36 assessment, the intervention group significantly outperformed the control group in the dimensions of physical functioning (β = 6.07, *p* = 0.031), social functioning (β = 3.85, *p* = 0.027) and mental health (β = 6.51, *p* = 0.013). The dimensions of vitality (β = 6.60, *p* = 0.070) and physical role limitation (β = 5.95, *p* = 0.090) showed a trend toward near significance. Although some variables did not reach statistical significance, the direction of the associations remained consistent with the primary outcomes.

**Table 8.** **Association between intervention group and post-treatment outcomes after adjustment for baseline covariates**.

	β (EEG-BF vs. Control)	*p* value
MoCA	0.77	0.072
PSQI	–0.83	0.030
SAS	–3.22	0.002
SDS	–2.49	0.002
Physical functioning	6.07	0.031
Physical role limitation	5.95	0.090
Vitality	6.60	0.070
Social functioning	3.85	0.027
Mental health	6.51	0.013

EEG-BF, electroencephalographic biofeedback; MoCA, Montreal Cognitive Assessment; PSQI, Pittsburgh Sleep Quality Index; SAS, Self-rating Anxiety Scale; SDS, Self-rating Depression Scale.

## Discussion

VCI-ND has been reported to be an important factor contributing to the progression of patients to dementia, but no effective treatment can be applied for the disease at present, and non-pharmacological therapies have been recognised as therapeutic modalities that may have potential [4, 26]. The results of the present study suggest that EEG-BF as a non-pharmacological intervention may play a beneficial role in patients with VCI-ND. After controlling for potential confounding factors by using PSM, the EEG-BF group showed a trend of better improvement than the control group in several key indicators, including cognitive function, sleep quality, anxiety and depression levels and quality of life. In particular, statistically significant improvements were noted in the scores of MoCA, SDS, SAS and some of the SF-36-dimensions. This finding suggests that EEG-BF is not only valuable in traditional mental disorders and ADHD interventions but may also be an important therapeutic strategy for the early intervention of VCI-ND. Notably, the study found that the standard treatment modality had limited effects on improving cognitive function, sleep quality and quality of life in patients with VCI-ND. This finding is consistent with those of previous studies, underscoring the need for more effective interventions [27].

From the perspective of cognitive improvement, EEG-BF may promote self-organisation and plasticity regulation of cortical neural networks by modulating EEG rhythms, thereby improving attention, working memory and information integration efficiency [28, 29]. Previous studies and reviews have shown that EEG-BF can improve cognitive and behavioural performance in the early stages of MCI and ADHD by increasing SMR with low β activity [10, 30]. In the present case, the EEG-BF group showed a significant increase in MoCA scores. In addition, previous studies have suggested that neurofeedback training may enhance functional connectivity in brain regions associated with cognitive control and attention, which are frequently affected by vascular lesions in VCI-ND [31, 32]. Although EEG-BF studies in patients with VCI-ND remain few, the above findings provide a theoretical basis for future relevant neuromodulatory interventions in this population. Given the lack of pharmacological treatments proven effective for VCI-ND, these findings highlight the potential of EEG-BF as a safe, non-invasive neuromodulatory approach to support cognitive rehabilitation in this population. A notable detail that in the present study, some baseline variables, including per capita household income, differed significantly between the two groups after PSM. Further sensitivity analyses indicated that these residual confounders may have influenced the effect of EEG-BF on cognitive improvement to some extent. This finding suggests that the robustness of EEG-BF’s intervention effect needs to be verified in larger samples and prospective studies.

In terms of mood improvement, EEG-BF training helps lower limbic system excitability and anxiety response thresholds. Previous studies have shown that α training or EEG-BF therapy is effective in alleviating mood problems in various disorders such as generalised anxiety disorder and insomnia comorbid with anxiety [13, 33]. The present study similarly found that the EEG-BF group showed significant improvement in SAS and SDS scores compared with the control group, and this finding may be attributed to the EEG-BF reward mechanism during training that reshaped individuals’ perceptual responses to stressors [13]. The high prevalence of mood disorders in patients with VCI-ND has been reported to be closely associated with chronic inflammation, white matter lesions, and serum cortisol abnormalities, and EEG-BF provides an effective non-pharmacological modulation pathway that is expected to be an important adjunctive treatment modality in this population [34, 35].

Sleep improvement was one of the main benefits of the EEG-BF group in this study. Sleep disorders are prevalent in VCI and may further exacerbate cognitive deterioration through disturbed sleep ratios, sleep deprivation and rhythm disturbances [3, 36]. EEG-BF have shown promising results in chronic insomnia, anxiety-related insomnia and sleep interventions in athletes [15, 37]. In the present study, the PSQI scores of the EEG-BF group decreased after treatment, and although the post-intervention difference with the control group was only close to the significant level, it tentatively suggests an excellent potential for improvement with the current limited duration and intensity of the intervention. The mechanism of this effect may involve rhythmic remodelling of thalamo-cortical circuit function and stabilising the regulation of autonomic activity. These findings highlight the potential of EEG-BF as a non-pharmacological strategy for managing sleep disturbances in VCI-ND, and suggest that it may contribute to slowing disease progression through improved sleep regulation.

The improvement in quality of life is another important finding of this study, demonstrating the combined effect of EEG-BF in improving cognitive, emotional and physiological functions. Compared with the control group, the EEG-BF group showed significant improvement in several dimensions of SF-36, suggesting that EEG-BF had a positive impact on patients’ quality of life. The above findings are consistent with those of previous studies involving patients with traumatic brain injury and post-traumatic stress disorder [38, 39]. Considering that the VCI-ND population is often in a borderline state of declining social functioning and is able to delay the decline in their quality of life, EEG-BF is believed to have a real-world significance in delaying the progression of dementia that should not be overlooked. Notably, the effect of EEG-BF intervention on the improvement of some dimensions of quality of life (e.g., physical pain) was not significant. This result may be related to the limited modulation of pain pathways by EEG-BF in patients with VCI-ND, suggesting that its benefits in emotional, cognitive, and other functions may be stronger than the effect of the intervention on somatic symptoms. This finding underscores the need for more targeted and symptom-specific neurofeedback protocols to fully leverage the benefits of EEG-BF across different domains of functioning.

This study found that EEG-BF therapy has several advantages over conventional treatment modalities when applied to patients with VCI-ND. Firstly, the therapy offers a potentially viable option for the VCI-ND patient population that lacks effective conventional drug therapy. Secondly, the therapy can be individually tailored to the training content and target frequency bands, and it has good plasticity and compliance, which can realise personalised medicine.

Several limitations should be acknowledged. Firstly, this study was a single-centre retrospective study, which may have led to selection bias. Although PSM was used to control for some confounding factors, and most covariates achieved good balance after matching, some variables still showed residual imbalance with SMDs exceeding the commonly accepted threshold of 0.2; Future research should be tailored to potential confounders [40]. Considering that multiple regression analyses were conducted across several SF-36 subscales without applying multiple comparison correction, the risk of type I error may be increased. Therefore, these findings should be interpreted with caution and warrant confirmation in future studies. Given the retrospective design of this study, the sample size was based on the actual number of cases and not estimated ex ante. Post-hoc efficacy analyses were performed using GPower software, which showed that the detection efficacy for the actual sample size (59 cases per group) was 0.768 with a medium effect size (Cohen’s d = 0.5) and α = 0.05. The effect size is slightly lower than the traditional standard (0.80) but is still of value in exploratory studies. Secondly, the intervention period in this study was 12 weeks, and the long-term sustained effect of EEG-BF therapy deserves further investigation. In addition, although internationally recognised scales, such as MoCA, PSQI, SAS and SDS with SF-36, were included, the support of objective brain functional imaging or neurophysiological data and corresponding mechanism studies was still lacking. Future multicentre prospective trials incorporating brain imaging and physiological monitoring are needed to further validate the efficacy and underlying mechanisms of EEG-BF therapy. Finally, changes in vascular risk factors during the intervention period in patients may have had some confounding effect on outcome indicators and need to be controlled for subsequent studies.

## Conclusion

The results of this study tentatively support that EEG-BF therapy, as an effective non-pharmacological intervention, may help to improve cognitive function, mood state, sleep quality and quality of life in patients with VCI-ND. Although the current evidence is insufficient, this study found that EEG-BF shows promising application in this population and warrants future clinical trials with larger samples and higher quality. Future studies are warranted to compare EEG-based biofeedback with other non-pharmacological interventions, such as cognitive training or rehabilitation, to clarify its unique therapeutic advantages and optimise individualised treatment strategies.

## Availability of Data and Materials

All experimental data included in this study can be obtained by contacting the corresponding author if needed.
